# Relationship between insomnia and cognitive function in elderly Chinese cerebral small vessel disease patients: mediating effect of neutrophil-to-lymphocyte ratio

**DOI:** 10.3389/fpsyt.2026.1876232

**Published:** 2026-08-12

**Authors:** Xiaoming Guo, Zhiyong Cao, Zi Zhuang, Can Xing, Yan Li, Weiguan Chen, Xiangyang Zhu

**Affiliations:** 1 Department of Neurology, Nantong First People’s Hospital, Nantong, China; 2 Department of Rehabilitation Medicine, Nantong First People’s Hospital, Nantong, China

**Keywords:** cerebral small vessel disease, cognitive function, insomnia, mediating effect, neutrophil-to-lymphocyte ratio, sleep parameters

## Abstract

**Background:**

This exploratory study investigated the relationship between insomnia, neutrophil-to-lymphocyte ratio (NLR), and cognitive function in elderly Chinese cerebral small vessel disease (CSVD) patients by integrating subjective and objective sleep assessments, and to analyze the mediating effect of NLR.

**Methods:**

A total of 86 CSVD patients aged ≥ 60 years were enrolled. Sleep was evaluated by polysomnography (PSG) and Pittsburgh Sleep Quality Index (PSQI). Cognitive function was evaluated by Montreal Cognitive Assessment (MoCA). Partial correlation analysis was used to investigate correlations between variables. Regression analysis and the bootstrap method were performed to investigate the mediating effect of NLR between sleep parameters and cognitive function. All models were adjusted for age, gender, years of education, and total CSVD burden.

**Results:**

Of the 86 patients, 50 (58.1%) had insomnia and 56 (65.1%) had CSVD-related cognitive impairment (CSVD-CI). Partial correlation analysis revealed that PSQI scores, wake after sleep onset (WASO), and arousal index (ArI) were positively correlated with NLR and negatively correlated with MoCA scores, whereas N3 proportion and sleep efficiency (SE) showed the opposite correlations; NLR was negatively correlated with MoCA scores (*P* < 0.05 for all). Mediation analysis further revealed that NLR partially mediated associations between the five sleep parameters (PSQI scores, N3, SE, WASO, ArI) and MoCA scores. All indirect effects were statistically significant, as the 95% bootstrap CIs did not include zero. The mediation proportions ranging from 27.50% to 33.72%.

**Conclusion:**

Insomnia is associated with cognitive impairment in elderly Chinese CSVD patients, a relationship that is partially mediated by elevated NLR.

## 1 Introduction

Cerebral small vessel disease (CSVD) is one of the primary causes of vascular cognitive impairment and dementia in the elderly (1–3). The characteristic imaging markers for CSVD include lacunes, white matter hyperintensities (WMH), cerebral microbleed (CMB), and enlarged perivascular spaces (EPVS) (4). With the acceleration of population aging in China, the burden of CSVD is becoming increasingly heavy, and the number of individuals affected by CSVD-related cognitive impairment (CSVD-CI) continues to rise (3), thus posing a serious challenge to public health. Therefore, identifying effective strategies for the prevention and intervention of CSVD-CI is of great practical significance for delaying cognitive decline and promoting active and healthy aging.

Sleep plays a vital role in maintaining cognitive function (5). However, sleep disorders, particularly insomnia, are highly prevalent in the elderly population (6), and often coexist with CSVD. Studies have shown that the proportion of CSVD patients meeting the diagnostic criteria for chronic insomnia is as high as 54%, significantly higher than that in healthy elderly individuals (7). Consequently, the specific relationship between insomnia and cognitive function is of particular concern in the CSVD population. Previous studies have confirmed that insomnia is associated with cognitive decline in CSVD patients (8), suggesting insomnia may be a key modifiable factor underlying the onset and progression of CSVD-CI.

Neutrophil-to-lymphocyte ratio (NLR), an inflammatory index derived from routine peripheral blood tests, is easily obtainable, objective, and reliable, and is considered to be an effective indicator of subclinical inflammatory status (9, 10). An elevated NLR typically indicates higher neutrophil and lower lymphocyte counts, reflecting a dynamic imbalance between innate immunity (neutrophil) and adaptive immunity (lymphocyte) (11).

Sleep plays a key role in immune regulation. Long-term or severe sleep disturbances, such as insomnia, can activate the hypothalamus-pituitary-adrenal (HPA) axis and the sympathetic nervous system (SNS), thus disrupting immune balance and triggering inflammatory responses (12). Consistent with this mechanistic framework, clinical studies have confirmed that patients with chronic insomnia exhibit significantly elevated NLR, a peripheral biomarker reflecting sleep-induced inflammatory status and immune imbalance (13). Furthermore, several studies have shown that NLR is not only an important risk factor for CSVD (10, 14, 15) but also significantly associated with an increased risk of CSVD-CI (16–18). Thus, NLR is known to be closely associated with both insomnia and CSVD-CI. It is biologically plausible that insomnia-triggered NLR elevation mediates the link between insomnia and cognitive decline via systemic inflammation-mediated cerebral small vessel injury. However, the inter-relationships between these three factors have yet to be fully elucidated. In addition, previous studies have predominantly relied on subjective scales to evaluate sleep and lacked a comprehensive analysis integrating objective polysomnography (PSG) parameters.

Therefore, in this study, we employed a combination of subjective and objective sleep assessments to systematically investigate intrinsic associations between insomnia, NLR, and cognitive function in elderly CSVD patients, and to further verify the mediating effect of NLR on the relationship between insomnia and cognitive function. Our goal was to provide a scientific theoretical basis and clinical reference for delaying cognitive decline and developing targeted comprehensive interventional strategies for elderly CSVD patients.

## 2 Materials and methods

### 2.1 Participants

This cross-sectional study included 120 consecutive patients aged 60 years or older with CSVD from the outpatient clinics or inpatient wards of the Department of Neurology, Nantong First People’s Hospital, between October 2024 and December 2025. Each patient underwent MRI and was identified as having at least one typical CSVD marker according to strict imaging criteria: lacunes, WMH, CMBs, or EPVS (4). Insomnia was diagnosed according to the chronic insomnia criteria described in the International Classification of Sleep Disorders, Third Edition (ICSD-3) (19).

**Figure 1:**
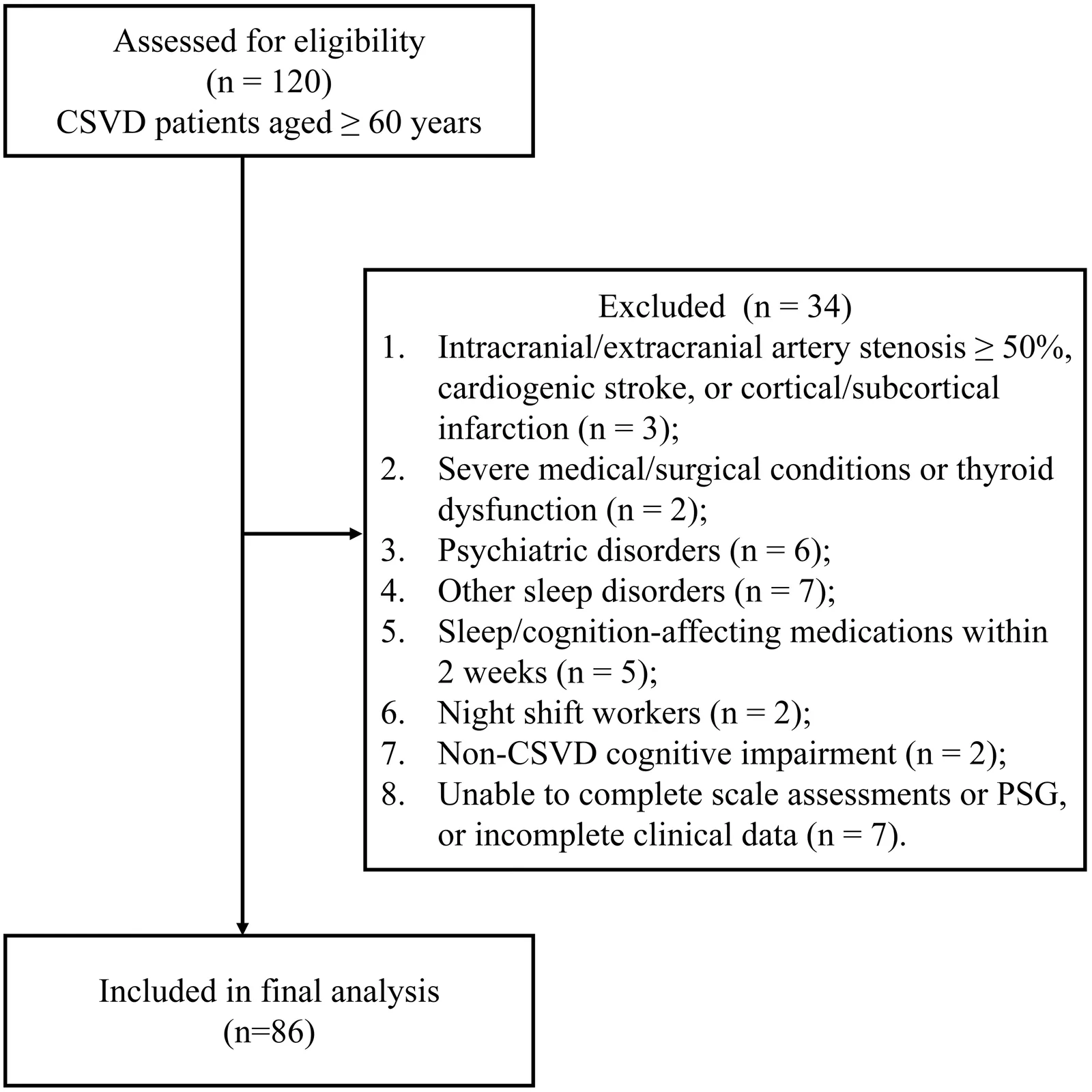
Participant screening flowchart.

Of the 120 patients, we excluded the following patients: (1) patients with intracranial or extracranial artery stenosis ≥ 50%, cardiogenic stroke, or cortical/subcortical infarction (*n* = 3); (2) patients with severe medical or surgical conditions, or thyroid dysfunction (*n* = 2); (3) patients with concurrent functional psychiatric disorders, including anxiety and a Hamilton Anxiety Scale (HAMA) score ≥ 14 or depression with a 17-item Hamilton Depression Rating Scale (HAMD-17) score > 7 (*n* = 6); (4) patients with other types of sleep disorders, such as sleep-disordered breathing, restless legs syndrome, or periodic limb movement disorder (*n* = 7); (5) patients who had used any medication affecting sleep or cognitive function within the previous two weeks (*n* = 5); (6) night shift workers (n = 2); (7) patients with cognitive impairment due to causes other than CSVD (*n* = 2); and (8) patients who were unable to cooperate with scale assessments or PSG examinations, or those with incomplete clinical data (*n* = 7). Finally, 86 patients were included (**Figure 1**). This study was approved by the Ethics Committee of Nantong First People’s Hospital (Reference: 2024KT596). Informed consent was obtained from all patients and their guardians.

### 2.2 Clinical data

From all participants, we collated a range of clinical data, including age, gender, years of education, body mass index (BMI), hypertension, diabetes, hyperlipidemia, smoking and alcohol drinking status.

### 2.3 Brain MRI scanning

Brain MRI scans were acquired using a 3.0 T MRI scanner with a 16-channel coil for brain imaging (Siemens, Germany) using the following sequences: axial T1-weighted imaging (T1WI), axial T2-weighted imaging (T2WI), axial diffusion-weighted imaging (DWI), coronal fluid-attenuated inversion recovery (FLAIR), and susceptibility-weighted imaging (SWI). The acquisition parameters were as follows: slice thickness = 5.0 mm, interslice gap = 1.5 mm, matrix size = 320 × 256, and field of view (FOV) = 240 × 240 mm². All images were independently evaluated by two experienced neurologists under double-blinded conditions according to published neuroimaging standards for CSVD (4). Disagreements were resolved by consensus. Lacunes were defined as round or oval lesions with diameters ranging from 3 to 15 mm and cerebrospinal fluid (CSF)-like signals. These lesions appeared hypointense on the T1WI sequence, hyperintense on the T2WI sequence, and hypointense in the center surrounded by a hyperintense rim on the FLAIR sequence. WMH were defined as round, oval, or patchy lesions that appeared hyperintense on the T2WI and FLAIR sequences and iso- or hypointense on the T1WI sequence. The severity of deep and periventricular WMH was assessed by Fazekas scoring, which ranged from 0 to 6. CMB were defined as small round or oval hypointense lesions with diameters between 2 and 5 mm on the susceptibility-weighted imaging (SWI) sequence. EPVS were defined as lesions generally < 3 mm in diameter with CSF-like signals. These appeared hypointense on the T1WI and FLAIR sequences, hyperintense on the T2WI, and were commonly located in the basal ganglia and centrum semiovale. EPVS were counted in the basal ganglia and centrum semiovale using a semi-quantitative scale, which is defined by grades 0 to 4: grade 0 (no EPVS), grade 1 (1–10 EPVS), grade 2 (11–20 EPVS), grade 3 (21–40 EPVS), and grade 4 (> 40 EPVS). With regards to the scoring of total CSVD burden, one point was assigned when any of the following features were present (20): lacune ≥ 1; moderate-to-severe WMH (Fazekas score 2–3 for deep WMH or score 3 for periventricular WMH); CMB ≥ 1; and moderate to severe (grade 2–4) EPVS in the basal ganglia. The total score was the sum of the points from all imaging markers and ranged from 0 to 4 points. A higher score indicated a more severe CSVD burden.

### 2.4 Sleep assessment

#### 2.4.1 PSG

Overnight PSG was conducted in the Sleep Monitoring Unit of the Department of Neurology, Nantong First People’s Hospital, using a Philips Alice 6 LDE PSG system. The monitoring room was sound-attenuated, light-controlled, and maintained at a temperature of approximately 18–26 °C. On the day of monitoring, participants were instructed to avoid daytime naps, strenuous exercise, alcohol, coffee, and strong tea. Electrode placement and technical parameters followed the standards outlined in the American Academy of Sleep Medicine (AASM) Manual for the Scoring of Sleep and Associated Events: Rules, Terminology and Technical Specifications (Version 2.6) (21). Sleep data were analyzed by trained and experienced physicians in accordance with AASM scoring criteria. The following objective sleep parameters were assessed: total sleep time (TST), sleep latency (SL), sleep efficiency (SE), wake after sleep onset (WASO), arousal index (ArI), and the proportion of total sleep time spent in each sleep stage [N1, N2, N3, and rapid eye movement (REM)].

#### 2.4.2 PSQI

Subjective sleep quality over the previous month was assessed in CSVD patients by a trained neurologist using the PSQI on the evening of the PSG monitoring. This scale consists of 19 self-rated items and 5 clinician-rated items, among which the 19^th^ self-rated item and the 5^th^ clinician-rated item were not included in scoring. The scale yields seven component scores: subjective sleep quality, SL, sleep duration, SE, sleep disturbances, use of sleeping medication, and daytime dysfunction. Each item is scored from 0 to 3 points, and the sum of all item scores yields the total PSQI score, ranging from 0 to 21 points. Higher scores indicate poorer sleep quality (22).

### 2.5 Cognitive function assessment

Cognitive function was assessed by a trained neurologist using the Beijing version of the MoCA on the evening of the PSG monitoring. The total MoCA score is 30 points, with a 1-point correction for those with ≤ 12 years of education. A score of < 26 was defined as cognitive impairment, in which a lower MoCA score indicates a greater severity of cognitive impairment (23).

### 2.6 Collection and measurement of NLR

Eligible patients underwent fasting venous blood collection on the morning after PSG monitoring (08:00-09:00). Absolute neutrophil and lymphocyte counts were determined using an automated hematology analyzer based on electrical impedance. The NLR was then calculated as the absolute neutrophil count divided by the absolute lymphocyte count.

### 2.7 Statistical analysis

Statistical analysis was performed using SPSS version 26.0 software. The Shapiro-Wilk’s test was used to test the normality of continuous variables. Continuous variables that followed a normal distribution are presented as mean ± standard deviation (SD) and were compared between groups using the independent-samples *t*-test. Continuous variables that did not follow a normal distribution are presented as median (interquartile range, IQR) and were compared using the Mann-Whitney *U* test. Categorical variables are presented as frequency and percentage [n (%)] and compared using the *χ²* test. Partial correlation analysis was employed to measure bivariate correlations between sleep parameters, NLR, and cognitive function. Baron and Kenny’s causal step approach (24), combined with PROCESS Macro Model 4 (Hayes, 2022) for SPSS (25), was used to investigate the relationship between sleep parameters, NLR, and cognitive function. For each sleep parameter, three models were constructed, estimating: (1) the effect of each sleep parameter on MoCA score; (2) the effect of each sleep parameter on NLR; and (3) the effect of each sleep parameter on MoCA score when NLR was controlled. Subsequently, mediation analysis was conducted using 5,000 bootstrap resamples to estimate total, direct, and indirect effects. An effect was considered statistically significant if its 95% confidence interval (CI) excluded zero; specifically, a significant mediating effect was indicated by a 95% CI for the indirect effect excluding zero. The proportion of mediation (PM) was calculated as (indirect effect/total effect) × 100%. All models were adjusted for age, gender, years of education, and total CSVD burden. Individual vascular risk factors were not separately adjusted, as the total CSVD burden comprehensively reflects cumulative microvascular damage induced by vascular risk exposures (20), thereby indirectly controlling for vascular confounding. Simultaneous adjustment for both vascular risk factors and total CSVD burden would introduce over-adjustment bias. To assess the robustness of the mediating effect of NLR under different covariate specifications, we performed stepwise sensitivity analyses. Model 1 was adjusted for age and years of education; Model 2 was additionally adjusted for gender; and Model 3 was further adjusted for total CSVD burden and hypertension. The significance and direction of the indirect effects were examined using a bootstrap procedure with 5,000 resamples. Multi-collinearity was assessed using the variance inflation factor (VIF), with a VIF < 10 indicating no significant multi-collinearity. A two-tailed P value < 0.05 was considered statistically significant.

## 3 Results

### 3.1 Comparison of clinical characteristics between the insomnia and non-insomnia groups

A total of 86 CSVD patients were enrolled. The mean age was 67.93 ± 5.50 years, and 48 (55.8%) were female. Of these patients, 50 (58.1%) were assigned to the insomnia group, and 36 (41.9%) were assigned to the non-insomnia group. No statistically significant differences were observed between the two groups in terms of age, years of education, BMI, history of hypertension, diabetes, hyperlipidemia, smoking, and alcohol consumption (*P* > 0.05 for all). Compared with the non-insomnia group, the insomnia group featured a higher proportion of females (*P* = 0.025), a higher NLR (*P* = 0.022), a higher total CSVD burden (*P* = 0.032), a higher proportion of cognitive impairment (*P* = 0.013), and lower MoCA scores (*P* < 0.001). With regards to sleep parameters, the insomnia group exhibited higher PSQI scores (*P* < 0.001), a shorter TST (*P* < 0.001), a longer SL (*P* < 0.001), a lower SE (*P* < 0.001), a longer WASO (*P* < 0.001), a higher ArI (*P* = 0.002), an increased N1 proportion (*P* < 0.001), and reduced N3 (*P* < 0.001) and REM proportions (*P* = 0.008), as shown in **Table 1**.

**Table 1:** Comparison of clinical characteristics between the insomnia and non-insomnia groups.

Variables	Insomnia group (n = 50)	Non-insomnia group (n = 36)	*t/χ²/Z*	*P*
Age, year, mean ± SD	68.80 ± 5.97	66.72 ± 4.57	1.750	0.084
Female, n (%)	33 (66.0)	15 (41.7)	5.025	0.025
Years of education, year, mean ± SD	9.64 ± 2.30	10.42 ± 2.35	−1.531	0.129
BMI, kg/m^2^, mean ± SD	24.97 ± 3.35	24.24 ± 2.72	1.079	0.284
Hypertension, n (%)	34 (68.0)	19 (52.8)	2.051	0.152
Diabetes, n (%)	18 (36.0)	9 (25.0)	1.176	0.278
Hyperlipidemia, n (%)	21 (42.0)	13 (36.1)	0.304	0.582
Smoking, n (%)	12 (24.0)	11 (30.6)	0.459	0.498
Drinking, n (%)	17 (34.0)	15 (41.7)	0.527	0.468
Neutrophil count (×10^9^/L)	4.55 ± 1.82	3.78 ± 1.77	1.939	0.056
Lymphocyte count (×10^9^/L)	1.34 ± 0.47	1.59 ± 0.76	−1.908	0.060
NLR, median (IQR)	3.61 (2.08, 4.80)	2.42 (1.62, 3.46)	−2.298	0.022
Total CSVD burden, score, median (IQR)	2.00 (1.00, 3.00)	1.50 (1.00, 2.75)	−2.140	0.032
CI, n (%)	38 (76.0)	18 (50.0)	6.229	0.013
MoCA, score, mean ± SD	19.40 ± 6.57	23.75 ± 4.19	−3.744	<0.001
Sleep parameters				
PSQI, score, mean ± SD	10.28 ± 3.12	5.47 ± 2.41	7.725	<0.001
TST, min, mean ± SD	320.10 ± 48.29	392.10 ± 43.15	−7.134	<0.001
N1, %, mean ± SD	18.31 ± 3.85	15.10 ± 2.76	4.501	<0.001
N2, %, mean ± SD	66.57 ± 3.19	65.45 ± 4.04	1.444	0.152
N3, %, median (IQR)	2.95 (1.40, 4.23)	5.50 (3.00, 7.20)	−3.801	<0.001
REM, %, mean ± SD	12.13 ± 2.90	14.07 ± 3.69	−2.720	0.008
SL, min, mean ± SD	45.43 ± 13.75	28.53 ± 10.79	6.137	<0.001
SE, %, mean ± SD	69.45 ± 7.17	83.50 ± 5.49	−9.858	<0.001
WASO, min, mean ± SD	96.40 ± 35.35	49.32 ± 24.23	7.327	<0.001
ArI, times/h, mean ± SD	21.65 ± 8.78	16.10 ± 6.46	3.219	0.002

BMI, body mass index; NLR, neutrophil-to-lymphocyte ratio; CSVD, cerebral small vessel disease; CI, cognitive impairment; MoCA, Montreal Cognitive Assessment; PSQI, Pittsburgh Sleep Quality Index; TST, total sleep time; N1, N2, N3, REM, sleep stages; SL, sleep latency; SE, sleep efficiency; WASO, wake after sleep onset; ArI, arousal index.

### 3.2 Comparison of clinical characteristics between the CSVD-CI and CSVD-NC groups

Of the 86 CSVD patients, 56 (65.1%) were assigned to the CSVD with cognitive impairment (CSVD-CI) group and 30 (34.9%) to the CSVD with normal cognition (CSVD-NC) group. No statistically significant differences were detected between the two groups in terms of gender, BMI, history of hypertension, diabetes, hyperlipidemia, smoking, or alcohol consumption (*P* > 0.05 for all). However, compared with the CSVD-NC group, the CSVD-CI group was older (*P* < 0.001), had fewer years of education (*P* = 0.001), and exhibited a higher NLR (*P* = 0.009), a higher total CSVD burden (*P* < 0.001), and a higher proportion of insomnia (*P* = 0.013). With regards to sleep parameters, the CSVD-CI group exhibited higher PSQI scores (*P* = 0.004), a shorter TST (*P* = 0.006), a lower SE (*P* = 0.001), a longer WASO (*P* < 0.001), a higher ArI (*P* = 0.004), an increased N1 proportion (*P* < 0.001), and reduced N3 (*P* < 0.001) and REM proportions (*P* = 0.043), as shown in **Table 2**.

**Table 2:** Comparison of clinical characteristics between the CSVD-CI and CSVD-NC groups.

Variables	CSVD-CI group (n = 56)	CSVD-NC group (n = 30)	*t/χ²/Z*	*P*
Age, year, mean ± SD	69.43 ± 5.66	65.13 ± 3.92	4.125	<0.001
Female, n (%)	32 (57.1)	16 (53.3)	0.115	0.735
Years of education, year, mean ± SD	9.36 ± 2.25	11.10 ± 2.09	−3.506	0.001
BMI, kg/m^2^, mean ± SD	25.02 ± 3.20	24.01 ± 2.86	1.455	0.149
Hypertension, n (%)	38 (67.9)	15 (50.0)	2.634	0.105
Diabetes, n (%)	20 (35.7)	7 (23.3)	1.390	0.238
Hyperlipidemia, n (%)	25 (44.6)	9 (30.0)	1.752	0.186
Smoking, n (%)	14 (25.0)	9 (30.0)	0.249	0.618
Drinking, n (%)	22 (39.3)	10 (33.3)	0.296	0.586
Neutrophil count (×10^9^/L)	4.49 ± 1.97	3.73 ± 1.42	1.872	0.065
Lymphocyte count (×10^9^/L)	1.35 ± 0.64	1.61 ± 0.55	−1.900	0.061
NLR, median (IQR)	3.65 (2.12, 4.96)	2.43 (1.60, 3.08)	−2.605	0.009
Total CSVD burden, score, median (IQR)	2.00 (2.00, 3.00)	1.00 (1.00, 2.00)	−5.544	<0.001
Insomnia, n (%)	38 (67.9)	12 (40.0)	6.229	0.013
MoCA, score, mean ± SD	18.36 ± 5.65	26.57 ± 1.33	−10.353	<0.001
Sleep parameters				
PSQI, score, mean ± SD	9.09 ± 3.60	6.73 ± 3.43	2.936	0.004
TST, min, mean ± SD	337.91 ± 59.34	373.35 ± 49.11	−2.797	0.006
N1, %, mean ± SD	17.99 ± 3.85	15.04 ± 2.77	3.705	<0.001
N2, %, mean ± SD	66.41 ± 3.62	65.52 ± 3.53	1.095	0.276
N3, %, median (IQR)	2.85 (1.50, 4.30)	5.05 (3.25, 7.03)	−3.635	<0.001
REM, %, mean ± SD	12.41 ± 3.20	13.94 ± 3.51	−2.052	0.043
SL, min, mean ± SD	39.94 ± 14.92	35.40 ± 15.17	1.339	0.184
SE, %, mean ± SD	72.82 ± 9.56	80.01 ± 7.58	−3.559	0.001
WASO, min, mean ± SD	86.56 ± 40.05	58.26 ± 28.96	3.762	<0.001
ArI, times/h, mean ± SD	21.18 ± 8.69	15.87 ± 6.38	2.942	0.004

BMI, body mass index; NLR, neutrophil-to-lymphocyte ratio; CSVD, cerebral small vessel disease; CI, cognitive impairment; NC, normal cognition; MoCA, Montreal Cognitive Assessment; PSQI, Pittsburgh Sleep Quality Index; TST, total sleep time; N1, N2, N3, REM, sleep stages; SL, sleep latency; SE, sleep efficiency; WASO, wake after sleep onset; ArI, arousal index.

### 3.3 Partial correlation analysis of sleep parameters, NLR, and cognitive function

As shown in **Table 3**, after adjusting for age, gender, years of education, and total CSVD burden, we identified that PSQI scores, WASO, and ArI were all positively correlated with NLR levels (PSQI: *r* = 0.293, *P* = 0.008; WASO: *r* = 0.347, *P* = 0.001; ArI: *r* = 0.346, *P* = 0.001) and negatively correlated with MoCA scores (PSQI: *r* = –0.371, *P* = 0.001; WASO: *r* = –0.369, *P* = 0.001; ArI: *r* = –0.386, *P* < 0.001). Furthermore, N3 proportion and SE were negatively correlated with NLR levels (N3: *r* = –0.306, *P* = 0.005; SE: *r* = –0.285, *P* = 0.009) and positively correlated with MoCA scores (N3: *r* = 0.396, *P* < 0.001; SE: *r* = 0.352, *P* = 0.001). In addition, a negative correlation was detected between NLR levels and MoCA scores (*r* = –0.445, *P* < 0.001).

**Table 3:** Results of partial correlation analysis of sleep parameters, NLR, and cognitive function.

Variables	NLR		MoCA	
	*r*	*P*	*r*	*P*
PSQI	0.293	0.008	−0.371	0.001
TST	−0.103	0.356	0.135	0.227
N1	0.157	0.160	−0.211	0.057
N2	0.129	0.248	−0.160	0.151
N3	−0.306	0.005	0.396	<0.001
REM	−0.077	0.490	0.103	0.358
SL	0.075	0.503	−0.194	0.081
SE	−0.285	0.009	0.352	0.001
WASO	0.347	0.001	−0.369	0.001
ArI	0.346	0.001	−0.386	<0.001
NLR	—	—	−0.445	<0.001

r, partial correlation coefficient.

PSQI, Pittsburgh Sleep Quality Index; MoCA, Montreal Cognitive Assessment; NLR, neutrophil-to-lymphocyte ratio; WASO, wake after sleep onset; ArI, arousal index; TST, total sleep time; SL, sleep latency; SE, sleep efficiency; N1, N2, N3, REM, sleep stages.

### 3.4 Mediating effect of NLR on the relationship between sleep parameters and cognitive function

Regression and mediation analyses were performed to investigate the relationships between the five key sleep parameters (PSQI, N3, SE, WASO, ArI) and cognitive function, with a separate model constructed for each sleep parameter. All models were adjusted for age, gender, years of education, and total CSVD burden. Multi-collinearity diagnostics revealed that the VIF was < 10, indicating no significant multi-collinearity.

As shown in **Table 4**, in Model 1, PSQI scores, WASO, and ArI were significant negative predictors of MoCA scores (PSQI: *β* = −0.247, *P* = 0.001; WASO: *β* = −0.261, *P* = 0.001; ArI: *β* = −0.264, *P* < 0.001), whereas N3 proportion and SE were significant positive predictors of MoCA scores (N3: *β* = 0.280, *P* < 0.001; SE: *β* = 0.251, *P* = 0.001).

**Table 4:** Regression analysis models for the mediating role of NLR between sleep parameters and cognitive function.

Variables	Model 1: cognitive function		Model 2: NLR		Model 3: cognitive function	
	*β* (95% CI)	*P*	*β* (95% CI)	*P*	*β* (95% CI)	*P*
PSQI	−0.247 (−0.385, −0.109)	0.001	0.281 (0.070, 0.485)	0.008	−0.175 (−0.309, −0.041)	0.011
NLR					−0.256 (−0.396, −0.116)	<0.001
N3	0.280 (0.135, 0.425)	<0.001	−0.311 (−0.527, −0.096)	0.005	0.203 (0.061, 0.345)	0.006
NLR					−0.249 (−0.388, −0.109)	0.001
SE	0.251 (0.102, 0.400)	0.001	−0.292 (−0.511, −0.074)	0.009	0.175 (0.031, 0.319)	0.018
NLR					−0.261 (−0.402, −0.120)	<0.001
WASO	−0.261 (−0.406, −0.115)	0.001	0.351 (0.140, 0.563)	0.001	−0.173 (−0.318, −0.027)	0.021
NLR					−0.251 (−0.395, −0.107)	0.001
ArI	−0.264 (−0.404, −0.124)	<0.001	0.340 (0.135, 0.544)	0.001	−0.180 (−0.320, −0.040)	0.013
NLR					−0.246 (−0.389, −0.103)	0.001

Model 1: total effect (path c); Model 2: effect of sleep parameters on NLR (path a); Model 3: direct effect (path c′) after adjustment for NLR, plus the effect of NLR on cognitive function (path b). β, standardized regression coefficient; CI, confidence interval.

MoCA, Montreal Cognitive Assessment; NLR, neutrophil-to-lymphocyte ratio; PSQI, Pittsburgh Sleep Quality Index; N3, slow-wave sleep; SE, sleep efficiency; WASO, wake after sleep onset; ArI, arousal index.

In Model 2, PSQI scores, WASO, and ArI were significant positive predictors of NLR (PSQI: *β* = 0.281, *P* = 0.008; WASO: *β* = 0.351, *P* = 0.001; ArI: *β* = 0.340, *P* = 0.001), while N3 proportion and SE were significant negative predictors of NLR (N3: *β* = −0.311, *P* = 0.005; SE: *β* = −0.292, *P* = 0.009).

After including the mediator NLR in Model 3, NLR was identified as a significant negative predictor of MoCA scores. The effect estimates of NLR for each model were as follows (PSQI model: *β* = −0.256, *P* < 0.001; N3 model: *β* = −0.249, *P* = 0.001; SE model: *β* = −0.261, *P* < 0.001; WASO model: *β* = −0.251, *P* = 0.001; ArI model: *β* = −0.246, *P* = 0.001). Compared with Model 1, the direct effects of each sleep parameter on MoCA scores were attenuated but remained statistically significant (PSQI: *β* = −0.175, *P* = 0.011; N3: *β* = 0.203, *P* = 0.006; SE: *β* = 0.175, *P* = 0.018; WASO: *β* = −0.173, *P* = 0.021; ArI: *β* = −0.180, *P* = 0.013).

**Figure 2:**
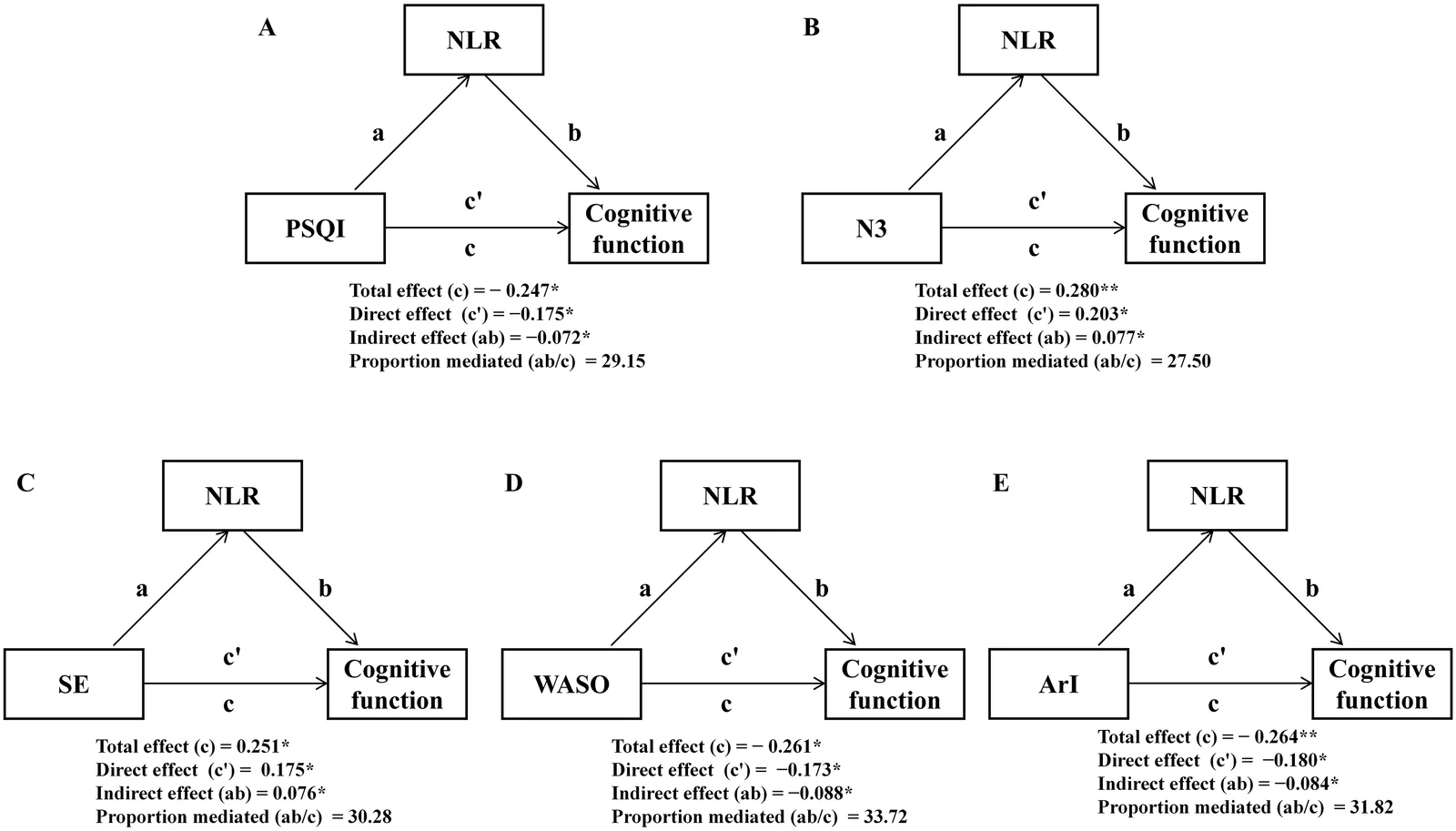
Path diagrams of the relationships between sleep parameters and cognitive function with NLR as a mediator. **(A)** PSQI; **(B)** N3; **(C)** SE; **(D)** WASO; **(E)** ArI. ^*^P < 0.05, ^**^P < 0.001. All models were adjusted for age, gender, years of education, and total CSVD burden. NLR, neutrophil-to-lymphocyte ratio; PSQI, Pittsburgh Sleep Quality Index; N3, slow-wave sleep; SE, sleep efficiency; WASO, wake after sleep onset; ArI, arousal index.

As shown in **Table 5** and **Figure 2**, bootstrap tests (5,000 resamples) revealed that total and direct effects remained statistically significant across all pathways. The indirect effect estimates for the five sleep parameters were as follows: PSQI (*β* = −0.072, 95% CI: −0.147 to −0.021), N3 (*β* = 0.077, 95% CI: 0.023 to 0.152), SE (*β* = 0.076, 95% CI: 0.015 to 0.153), WASO (*β* = −0.088, 95% CI: −0.176 to −0.022), and ArI (*β* = −0.084, 95% CI: −0.170 to −0.022). All indirect effects were statistically significant, as the 95% bootstrap CIs did not include zero. The corresponding mediation proportions were 29.15%, 27.50%, 30.28%, 33.72%, and 31.82%, respectively, ranking WASO > ArI > SE > PSQI > N3. These findings indicate that NLR partially mediated the relationships between sleep parameters and cognitive function.

**Table 5:** Bootstrap tests for the mediating effect of NLR between sleep parameters and cognitive function.

Model pathways	Effect type	*β*	Boot *SE*	*Bootstrap 95%* CI		PM, %
				Lower	Upper	
PSQI → NLR → Cognitive function	Total effect	−0.247	0.069	−0.385	−0.109	—
	Direct effect	−0.175	0.068	−0.309	−0.041	70.85
	Indirect effect	−0.072	0.032	−0.147	−0.021	29.15
N3 → NLR → Cognitive function	Total effect	0.280	0.073	0.135	0.425	—
	Direct effect	0.203	0.071	0.061	0.345	72.50
	Indirect effect	0.077	0.033	0.023	0.152	27.50
SE → NLR → Cognitive function	Total effect	0.251	0.075	0.102	0.400	—
	Direct effect	0.175	0.073	0.031	0.319	69.72
	Indirect effect	0.076	0.035	0.015	0.153	30.28
WASO → NLR → Cognitive function	Total effect	−0.261	0.073	−0.406	−0.115	—
	Direct effect	−0.173	0.073	−0.318	−0.027	66.28
	Indirect effect	−0.088	0.040	−0.176	−0.022	33.72
ArI → NLR → Cognitive function	Total effect	−0.264	0.070	−0.404	−0.124	—
	Direct effect	−0.180	0.071	−0.320	−0.040	68.18
	Indirect effect	−0.084	0.038	−0.170	−0.022	31.82

PM, proportion mediated (indirect effect/total effect × 100%); Boot SE, bootstrap standard error; CI, confidence interval.

PSQI, Pittsburgh Sleep Quality Index; NLR, neutrophil-to-lymphocyte ratio; N3, slow-wave sleep; SE, sleep efficiency; WASO, wake after sleep onset; ArI, arousal index.

Stepwise sensitivity analyses further verified the robustness of these pathways, with the indirect effects of all five sleep parameters mediated by NLR remaining statistically significant and directionally consistent across all covariate adjustment models (**Supplementary Table 1**).

## 4 Discussion

In this exploratory observational study, we investigated the associations between insomnia, NLR, and cognitive function in elderly CSVD patients using subjective and objective sleep parameters and revealed that NLR played a significant mediating role in the relationship between insomnia and cognitive function. Given the cross-sectional nature of our data, causal inferences were not permissible. Nevertheless, these findings provide a novel perspective for understanding the mechanisms underlying the association between insomnia and cognitive impairment in elderly CSVD patients.

Of the 86 CSVD patients in this study, the proportion with insomnia was 58.1%, thus suggesting that close attention should be paid to sleep screening in this clinical population. Furthermore, the proportion of insomnia was significantly higher in women than in men; this is consistent with the gender distribution of insomnia in the general population (26) and indicates that gender may represent an important factor influencing susceptibility to insomnia in CSVD patients. Both subjective and objective sleep assessments consistently identified multi-dimensional sleep disturbances in CSVD patients with insomnia. These patients reported significantly higher PSQI scores, and objectively exhibited shortened TST, prolonged SL, reduced SE, increased WASO, elevated ArI, and disrupted sleep architecture (increased N1 proportion, reduced N3 and REM proportions). Collectively, these results indicate that in CSVD patients with insomnia, subjective sleep quality is poor, and objective sleep structure and continuity are also markedly impaired. The mechanism by which CSVD may contribute to or exacerbate insomnia involves multiple factors, including hypoperfusion in sleep-regulating regions like the hippocampus (8), disruption of neural fiber tracts by white matter lesions that impairs the structural integrity and functional coordination of sleep-regulating networks (27–29), and chronic inflammation affecting sleep centers (30).

In the present study, we found that NLR levels were significantly higher in CSVD patients with insomnia than in those with normal sleep, which is consistent with the notion that chronic insomnia is accompanied by systemic inflammation (13). Furthermore, NLR was also significantly elevated in patients with CSVD-CI, further supporting a close association between NLR and CSVD-CI. Furthermore, CSVD patients with insomnia exhibited a higher incidence of cognitive impairment and poorer cognitive function, whereas CSVD-CI patients had a higher incidence of insomnia and more severe abnormalities in both subjective and objective sleep parameters. Collectively, these findings preliminarily suggest a close link between insomnia, elevated NLR, and cognitive decline in elderly CSVD patients.

To explore the sleep-inflammation-cognition pathway, we next performed a series of statistical analyses. Partial correlation analysis revealed that the deterioration of both subjective and objective sleep parameters (indicated by higher PSQI scores, a lower N3 proportion and SE, a longer WASO, and a higher ArI) was significantly correlated with elevated NLR. Concurrently, these sleep parameters, and an elevated NLR, were each significantly and independently correlated with cognitive decline (as indicated by lower MoCA scores). Regression and mediation analyses confirmed that these adverse sleep parameters were independent predictors of both cognitive decline and elevated NLR. Furthermore, NLR played a significant partial mediating role on the relationships between PSQI scores, N3 proportion, SE, WASO, ArI and cognitive function, with mediation effects ranging from 27.5% to 33.7%. These data suggest that systemic inflammatory responses may mediate approximately one-third of the association between insomnia and cognitive function in elderly CSVD patients.

Based on the existing literature, we propose the following hypothesis to contextualize our findings. Chronic insomnia is known to promote the release of pro-inflammatory cytokines via activation of the SNS and HPA axis (12, 31, 32). SE is a key indicator for the clinical evaluation of sleep quality in patients with insomnia (33). A reduced SE, prolonged WASO, and increased ArI all indicate sleep fragmentation, which can lead to the disruption of sleep architecture, particularly a reduction in deep sleep (N3 stage). Furthermore, N3 sleep is known to play a critical role in memory consolidation and restorative sleep (34). N3 deficiency can impair glymphatic system clearance, leading to the accumulation of neurotoxic substances including Aβ and tau proteins. This subsequently activates astrocytes and microglia, inducing neuroinflammation (35–38). In turn, neuroinflammation disrupts peripheral immune homeostasis *via* the neuro-immune circuit, promoting neutrophil activation and lymphocyte dysfunction, which ultimately elevates NLR (11, 39). This mechanism is supported by multiple previous studies. For instance, patients with chronic insomnia exhibit significantly elevated NLR (13). A previous study revealed that in patients with schizophrenia, SE is negatively correlated with neutrophil count and NLR, while WASO and awakening frequency are positively correlated with neutrophil count (40). Sleep fragmentation can lead to increased neutrophil and monocyte counts, accelerating atherosclerosis (41). Another study of hospitalized COVID-19 patients showed that patients with poor subjective sleep quality exhibited more pronounced lymphopenia and an elevated NLR (42). This evidence, spanning both subjective and objective measures, consistently supports a close association between sleep disturbances and an elevated NLR. As a marker of systemic inflammation, elevated NLR may further disrupt cognitive-related neural networks by exacerbating vascular endothelial injury and compromising the blood-brain barrier. This process may in turn aggravate CSVD-related microvascular lesions, reduce perfusion and metabolism in cognition-critical brain regions, impair synaptic function, and activate glial cells, ultimately exacerbating cognitive impairment in elderly CSVD patients (8, 10, 16, 18). In addition, NLR may be involved in the development and progression of cognitive dysfunction by regulating electroencephalographic activity (17).

Notably, two primary competing explanations exist for the cross-sectional associations observed in the analysis. First, cognitive dysfunction itself could disrupt sleep and raise inflammatory levels, rather than the reverse (43). Second, severe CSVD may serve as a shared confounder: extensive microvascular lesions simultaneously disturb sleep, trigger systemic inflammation, and impair cognitive networks, which may partially account for these cross-sectional relationships (44). In addition, N3 sleep exhibited the smallest mediating effect proportion (27.50%) among the five sleep parameters. This relatively low proportion does not diminish the biological significance of N3 sleep itself. Rather, it implies that N3 insufficiency may impair cognitive function *via* two separate but converging mechanistic routes: a peripheral inflammatory cascade quantified by NLR, and a direct central pathway driven by glymphatic insufficiency and neurotoxic protein deposition. While the respective magnitude of each route cannot be quantified with the present dataset, these two mechanisms are not mutually exclusive and may synergistically drive cognitive decline in CSVD patients.

This study has several limitations that need to be considered. First, the cross-sectional design of this research prevented us from confirming the causal relationships of the mediation effects; further validation in prospective studies is now required. Second, the single-center design and relatively small sample size may have limited statistical power and generalizability. Multi-center collaborative studies enrolling larger and more diverse cohorts are warranted to validate and extend our results. Third, while we adjusted for age, gender, education, and total CSVD burden, we did not control for vascular risk factors such as hypertension and diabetes. This decision aimed to avoid over-adjustment, as these factors may influence cognition indirectly *via* total CSVD burden. However, their exclusion may have overlooked direct effects that were independent of total CSVD burden. Future large-sample investigations are needed to disentangle the independent and mediating effects of distinct vascular risk factors. Fourth, as a non-specific peripheral inflammatory index, NLR may not fully reflect central neuroinflammatory status. In addition, using only MoCA for cognitive assessment may mask domain-specific impairments. Future studies should adopt a wider spectrum of inflammatory markers and comprehensive neuropsychological assessments covering multiple cognitive subdomains to systematically delineate systemic and cerebral inflammatory changes and their associated cognitive impacts. Fifth, a single-night PSG may be subject to the ‘first-night effect’; this effect has a limited impact on intergroup comparisons but may introduce bias in the absolute values of sleep parameters. Including an adaptation night prior to formal PSG recording in future research can mitigate this interference and yield more stable baseline sleep metrics. Finally, no multiple-testing correction was performed across the five independent sleep models, which may inflate Type I error. All sleep-cognition associations reported herein should be regarded as preliminary and require validation in separate external cohorts.

Despite these limitations, our findings have potential clinical implications. Our findings suggest that insomnia may adversely affect cognitive function by inducing or exacerbating systemic inflammation characterized by an elevated NLR, which plays a significant mediating role in this relationship. In clinical practice, clinicians could conduct routine sleep screening for elderly CSVD patients, using the PSQI as a practical first-line tool. Combining the PSQI with NLR may help identify patients at higher risk of cognitive decline, while objective PSG parameters could further aid risk stratification. A combined sleep-inflammation intervention strategy warrants further investigation: early sleep behavioral interventions may contribute to cognitive protection, and for CSVD-CI patients with insomnia, effective insomnia management may be associated with reduced NLR levels and slowed cognitive deterioration.

## Data Availability

The raw data supporting the conclusions of this article will be made available by the authors, without undue reservation.
