# Supplementary material for: Relationship between insomnia and cognitive function in elderly Chinese cerebral small vessel disease patients: mediating effect of neutrophil-to-lymphocyte ratio
Source: Front Psychiatry. 2026 Aug 12;17:1876232. doi: 10.3389/fpsyt.2026.1876232 (PMC13506932; doi:10.3389/fpsyt.2026.1876232)
Supplement: Supplementary file 1 [file Table1.docx]

Supplementary Material

| **Model pathways** | **Indirect effect** | ***β*** | **Boot *SE*** | ***Bootstrap 95%*CI** | | **PM, %** |
| --- | --- | --- | --- | --- | --- | --- |
|  |  |  |  | **Lower** | **Upper** |  |
| PSQI → NLR → Cognitive function | Model 1 | −0.136 | 0.049 | −0.247 | −0.058 | **41.74** |
|  | Model 2 | −0.130 | 0.050 | −0.243 | −0.049 | 39.98 |
|  | Model 3 | −0.063 | 0.030 | −0.136 | −0.016 | 27.08 |
| N3 → NLR → Cognitive function | Model 1 | 0.146 | 0.048 | 0.065 | 0.252 | **38.12** |
|  | Model 2 | 0.142 | 0.049 | 0.057 | 0.251 | 36.41 |
|  | Model 3 | 0.069 | 0.031 | 0.018 | 0.141 | 24.66 |
| SE → NLR → Cognitive function | Model 1 | 0.143 | 0.048 | 0.063 | 0.248 | **39.94** |
|  | Model 2 | 0.140 | 0.053 | 0.051 | 0.259 | 37.28 |
|  | Model 3 | 0.066 | 0.034 | 0.008 | 0.142 | 29.62 |
| WASO → NLR → Cognitive function | Model 1 | −0.156 | 0.057 | −0.287 | −0.066 | **38.75** |
|  | Model 2 | −0.153 | 0.057 | −0.279 | −0.055 | 37.62 |
|  | Model 3 | −0.078 | 0.039 | −0.168 | −0.015 | 33.52 |
| ArI → NLR → Cognitive function | Model 1 | −0.153 | 0.049 | −0.264 | −0.070 | **41.35** |
|  | Model 2 | −0.150 | 0.051 | −0.259 | −0.059 | 39.96 |
|  | Model 3 | −0.075 | 0.036 | −0.157 | −0.013 | 32.09 |

**Supplementary Table S1.** Stepwise sensitivity analyses of the mediating effect of NLR between sleep parameters and cognitive function

Note: Model 1: adjusted for age and years of education; Model 2: adjusted for age, gender, and years of education; Model 3: adjusted for age, gender, years of education, total CSVD burden, and hypertension; The primary mediation models in the main text were adjusted for age, gender, years of education, and total CSVD burden; PM, proportion mediated (indirect effect / total effect × 100%); Boot SE, bootstrap standard error; CI, confidence interval.

Abbreviations: NLR, neutrophil-to-lymphocyte ratio; PSQI, Pittsburgh Sleep Quality Index; N3, slow-wave sleep; SE, sleep efficiency; WASO, wake after sleep onset; ArI, arousal index.
